# Acute pancreatitis and obstructive jaundice as initial complaints of hepatocellular carcinoma: case report

**DOI:** 10.1186/1477-7819-12-13

**Published:** 2014-01-14

**Authors:** Guo-Qing Jiang, Jian-Jun Qian, Jie Yao, Sheng-Jie Jin, Dou-Sheng Bai

**Affiliations:** 1 Department of Hepatobiliary Surgery, Clinical Medical College of Yangzhou University, 98 West Nantong Road, Yangzhou 225000, Jiangsu Province, China

**Keywords:** Pancreatitis, Obstructive jaundice, Hepatocellular carcinoma, Common bile duct embolism

## Abstract

**Background:**

Patients with cirrhosis-associated hepatocellular carcinoma (HCC) rarely present with acute pancreatitis (AP) and obstructive jaundice as the main clinical features. AP with obstructive jaundice caused by common bile duct embolism (CBDE) is very rare.

**Case presentation:**

A 54-year-old man with CBDE was misdiagnosed with common bile duct stones three times over a 7-month period. Investigations during this time did not identify CBDE. Surgical exploration was performed because of AP, obstructive jaundice, and a tumor in the left lobe of the liver. CBDE from the hepatic tumor was diagnosed by intraoperative biopsy and frozen section examination. The patient underwent left hemihepatectomy, cholecystectomy, and bile duct exploration.

**Conclusion:**

Preoperative diagnosis of CBDE is difficult because of the rarity of the condition, lack of physician awareness, and easy misdiagnosis on imaging examinations. Early and accurate diagnosis of this condition is important.

## Background

Patients with cirrhosis-associated hepatocellular carcinoma (HCC) rarely present with acute pancreatitis (AP) and obstructive jaundice as the main clinical features. AP with obstructive jaundice caused by common bile duct embolism (CBDE) is very rare. Preoperative diagnosis of CBDE is difficult because of the rarity of the condition, lack of physician awareness, and easy misdiagnosis on imaging examinations. We report a rare case of CBDE causing AP with obstructive jaundice, to raise awareness of the importance of early and accurate diagnosis.

## Case presentation

**Figure 1:**
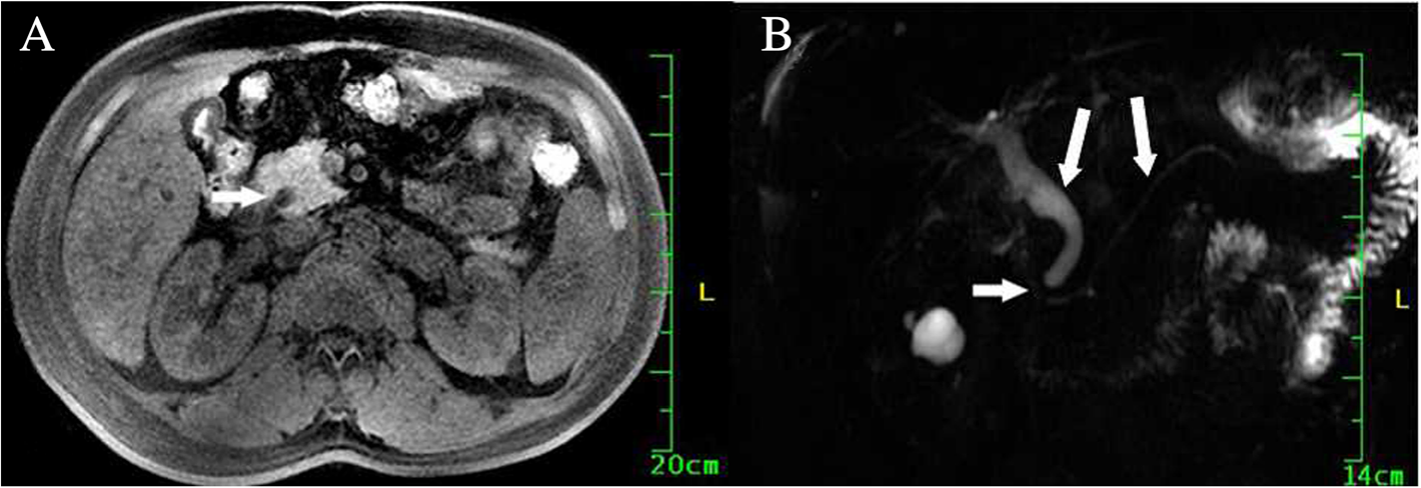
**Magnetic resonance imaging findings on 22 February 2011. (A)** Short inversion time inversion recovery image, showing a mildly dilated common bile duct. Heterogeneous isointense areas (white arrow) were observed in the distal portion of the common bile duct, suggesting common bile duct stones. **(B)** Magnetic resonance cholangiopancreatography, showing mild obstruction at the duodenal ampulla (short white arrow). The common bile duct and pancreatic duct were slightly dilated (long white arrows).

**Figure 2:**
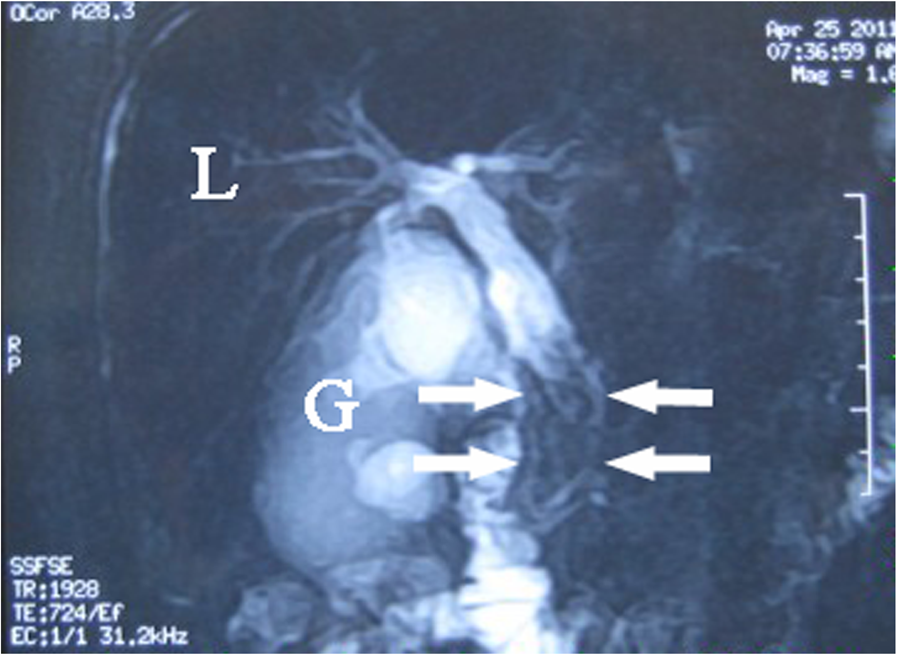
**Magnetic resonance imaging findings on 25 April 2011.** Magnetic resonance cholangiopancreatography, showing irregular hypointense areas in the common bile duct (white arrows) with obvious dilation of the distal bile ducts. G, gallbladder; L, liver.

A 54-year-old man with type 2 diabetes mellitus and liver cirrhosis secondary to hepatitis B virus infection was admitted to the Department of Gastroenterology at Clinical Medical College of Yangzhou University, Jiangsu Province, China, on 21 February 2011 because of abdominal pain. The patient had a 3-day history of yellow conjunctivae and urine. On 22 February 2011, abdominal magnetic resonance imaging (MRI) and magnetic resonance cholangiopancreatography (MRCP) showed a maximum common bile duct (CBD) diameter of 13 mm with an area of heterogeneous intensity signals in the distal portion of the CBD suggestive of CBD stones (Figure 1A), and normal intrahepatic bile ducts. There was mild obstruction at the duodenal ampulla (Figure 1B), liver cirrhosis, and enlargement of the spleen. Routine laboratory tests showed high serum levels of direct bilirubin (DBIL) (42.5 μmol/l, normal range 1.7 to 7.8 μmol/l), total bilirubin (TBIL) (62.7 μmol/l, normal range 5.7 to 23.5 μmol/l), aspartate aminotransferase (AST) (91 U/l, normal range 0 to 50 U/l), alanine aminotransferase (ALT) (206 U/l, normal range 0 to 50 U/l), and CA19-9 (821.03 U/ml, normal range <35 U/l); normal serum levels of amylase (62 U/l, normal range 30 to 110 U/l) and alpha-fetoprotein (AFP) (6.17 ng/ml, normal range 0 to 7 ng/ml); and a normal urinary amylase level (326 U/l, normal range 32 to 641 U/l). Endoscopic papillary balloon dilation (EPBD) was performed on 23 February 2011, and the procedure report described extraction of a muddy stone from the CBD. Eight days after EPBD, the plasma CA19-9 level was 469.56 U/ml, and hepatic function had improved with DBIL 10.6 μmol/l, TBIL 16.4 μmol/l, AST 36 U/l, and ALT (70 U/l). The report of a follow-up MRCP performed at another hospital, at a time when the patient was asymptomatic, described nodular cirrhosis, enlargement of the gallbladder, mild dilation of the intra- and extrahepatic bile ducts, irregular areas of low signal intensity in the CBD that were interpreted as CBD stones (Figure 2), and a 1.1 cm diameter mass with clear borders in the lateral portion of the left lobe of the liver that was hypointense on T1-weighted images and slightly hyperintense on T2-weighted images. The consulted team (surgeon, radiation oncologist, medical oncologist, and radiologist) considered that the hepatic mass might be a benign nodule, and should be monitored.

**Figure 3:**
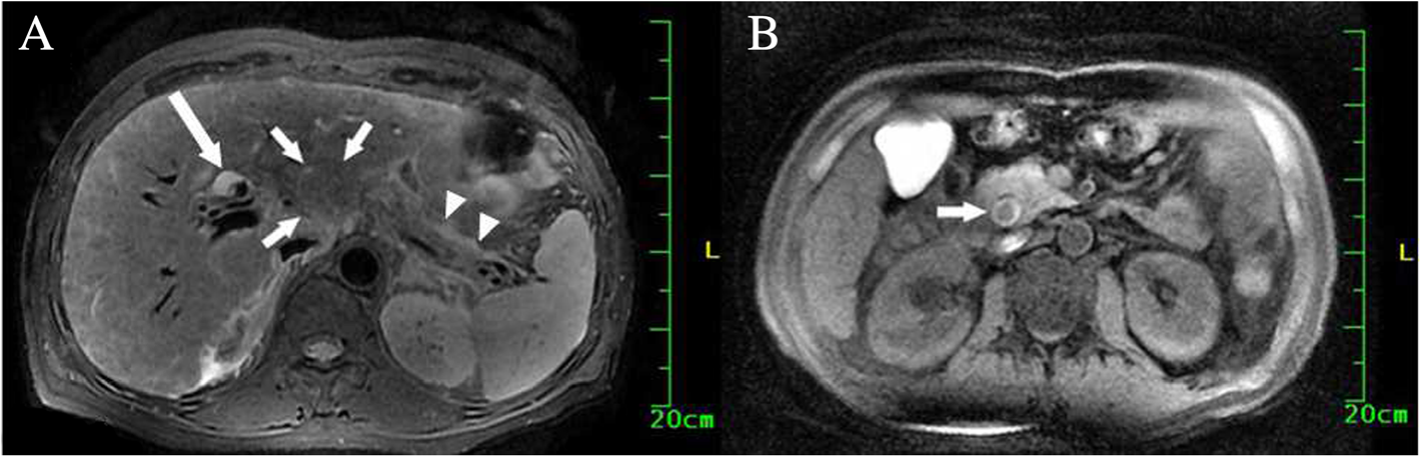
**Magnetic resonance imaging findings on 21 September 2011. (A)** Short inversion time inversion recovery image, showing an irregular, isointense mass with unclear margins (short white arrows) in the left lobe of the liver. There was fluid around the pancreas (white arrows) indicating pancreatitis, and mild dilation of the intrahepatic bile ducts (long white arrow). **(B)** Short inversion time inversion recovery image distal to the plane of image **(A)**, showing a dilated common bile duct with an isointense filling defect (white arrow).

The patient did not return to hospital until recurrence of pancreatitis 5 months later. He was hospitalized for AP and obstructive jaundice on 21 September 2011. On admission, abdominal ultrasonography showed liver cirrhosis with a 27 × 38 mm area of heterogeneous echogenicity in the left lobe and a thickened, uneven gallbladder containing muddy stones. The CBD was dilated (9 to 16 mm in diameter), with areas of dense, spotty echoes referred to as muddy stones, and the left intrahepatic bile duct was also slightly dilated. Abdominal MRI and MRCP on the day after admission showed cholecystitis, gallbladder stones, a mass in the left lobe of the liver with unclear margins that was isointense on short inversion time inversion recovery sequences (Figure 3A), filling defects in the CBD suggestive of stones (Figure 3B), dilation of the intra- and extrahepatic bile ducts, pancreatitis, and liver cirrhosis. On 5 October 2011, abdominal computed tomography (CT) showed an irregular, low-density mass measuring 27 × 38 mm in the left lobe of the liver, and a dilated CBD with muddy stones. Contrast-enhanced CT showed inhomogeneous enhancement of the liver mass and dilation of the intrahepatic bile ducts. The contrast density of the CBD stones was similar in the arterial and portal venous phases, ranging from 15 to 22 Hounsfield units (HU).

Routine laboratory tests showed high serum levels of amylase (1,423 U/l), DBIL (136.1 μmol/l), TBIL (141.2 μmol/l), AST (223 U/l), ALT (366 U/l), and CA19-9 (2041 U/ml); a high urinary amylase level (6,826 U/l); and a normal serum AFP level (6.36 ng/ml). The patient had acute epigastric pain, fever, and jaundice. The high serum amylase level and abdominal CT findings indicated a diagnosis of AP.

Because the pancreatitis was not severe and the patient’s condition gradually improved, emergency endoscopic retrograde cholangiopancreatography (ERCP) was not performed. After the pancreatitis was stabilized, surgical exploration of the abdomen was performed on 10 October 2011. Because of the patient’s history of liver cirrhosis secondary to hepatitis B virus infection, there was a high probability that the liver mass was hepatocellular carcinoma. We planned to perform cholecystectomy and bile duct exploration, with intraoperative diagnosis of the nature of the liver mass. Intraoperative exploration showed nodular cirrhosis of the liver, an edematous gallbladder, a large number of muddy gallbladder stones, and an unencapsulated tumor in the left lobe of the liver measuring 45 × 35 × 25 mm. A soft grayish-white embolus (45 × 11 × 9 mm) was extracted from the distal CBD, and grayish-white tumor debris was observed in the left intrahepatic bile duct. Intraoperative biopsy and frozen section examination showed HCC with tumor CBDE. Left hemihepatectomy, cholecystectomy, and bile duct exploration were performed. Postoperative pathological examination of the surgical specimen confirmed moderately differentiated HCC in the left lobe of the liver and the CBDE. The surgical margins were free of tumor cells.

The serum CA19-9 level decreased to 81.20 U/ml by the 13th day after surgery, and the patient was discharged from hospital on the 35th day after surgery. The patient subsequently died of liver failure in another hospital on 12 February 2012.

## Discussion

After reviewing the case, we consider that this patient was misdiagnosed prior to surgical exploration. The EPBD report describes extraction of muddy stones from the CBD. The report of the MRCP performed 61 days later also describes stones in the CBD. However, it is unlikely that so many muddy stones would have formed during this short time. Surgical exploration eventually revealed that the structures thought to be muddy stones were actually CBDE from HCC. These malignant emboli may have been misdiagnosed as muddy stones during EPBD because of their grayish-white, friable, loose appearance.

The factors that may have contributed to the initial misdiagnosis were: 1) absence of a visible liver mass in the early stage of disease; 2) a normal plasma AFP level; 3) similar morphological characteristics between tumor emboli and muddy stones, because of absence of a blood supply (arterial phase CT was unable to differentiate between tumor emboli and muddy stones); 4) the rarity of tumor emboli causing AP, compared with other causes of AP such as metabolic abnormalities, obesity, genetic susceptibility, and the two most common causes: gallstones and alcohol-related disease; and 5) cholecystitis associated with muddy stones in the gallbladder, leading to the assumption that AP was caused by stones in the CBD.

To our knowledge, there are very few reported cases of AP with obstructive jaundice caused by CBDE from cirrhosis-associated HCC. The cause of CBD obstruction was misdiagnosed three times, mainly because of the lack of experience of the clinicians with CBDE and the rarity of this condition. Preoperative diagnosis is difficult, because of the rarity of the condition, lack of physician awareness, and easy misdiagnosis on imaging examinations. However, the imaging tools available for diagnosis of HCC have recently improved.

At the time of the patient’s first visit, there was no sign of the liver mass on MRCP. We speculate that there was probably a small HCC at that time, which may have ruptured into or invaded the intrahepatic bile ducts, resulting in accumulation of tumor debris and emboli in the CBD. Buckmaster *et al.* reported HCC emboli in the CBD of a patient in whom no primary hepatic tumor was found on preoperative CT or surgical exploration [1]. If CBDE had been diagnosed in our patient at the first presentation, his HCC could have been treated earlier, resulting in longer survival. Misdiagnosis of CBDE should be avoided if possible. Our case illustrates the importance of early and accurate diagnosis. The common clinical features of this condition include liver cirrhosis secondary to viral hepatitis or other factors, high serum levels of AFP [2] and CA19-9 (in our case, the CA19-9 level was high but the AFP level was normal), history of cholangitis with dilation of the intra- and extrahepatic bile ducts, rapid development of liver dysfunction with jaundice, and a filling defect in the common bile duct of 2 cm or longer. If three or more of these features appear in the same patient, CBDE should be considered.

Contrast-enhanced CT findings are useful for differentiating between CBDE and a CBD stone. In our case, the contrast density of the CBDE was low in all phases of the scan, ranging from 15 to 22 HU, whereas the contrast density of CBD stones is usually >50 HU. When the contrast density of a filling defect in the CBD is ≤50 HU, CBDE should be considered.

The prognosis of this type of HCC is generally poor, but is better than for HCC with hepatocellular jaundice. Jaundice does not necessarily indicate advanced disease, and is not always a contraindication to surgery. Patients with HCC and obstructive jaundice due to CBDE may benefit greatly from surgery. Early and appropriate treatment of such patients may achieve satisfactory palliation, as well as occasional long-term resolution of symptoms and survival [3-5].

## Conclusion

In conclusion, we have described a very rare case of HCC presenting as AP with obstructive jaundice. Preoperative diagnosis is difficult, because of the rarity of the condition, lack of physician awareness, and easy misdiagnosis on imaging examinations. It is important to pay attention to the above-mentioned clinical data to obtain early and accurate diagnosis of this condition.

## Consent

Written informed consent was obtained from the patient for the publication of this case presentation and accompanying images. A copy of the written consent is available for review by the Editor-in-Chief of this journal.

## Abbreviations

AFP: Alpha-fetoprotein; ALT: Alanine aminotransferase; AP: Acute pancreatitis; AST: Aspartate aminotransferase; CA19-9: Carbohydrate antigen 19–9; CBD: Common bile duct; CBDE: Common bile duct embolism; CT: Computed tomography; DBIL: Direct bilirubin; EPBD: Endoscopic papillary balloon dilation; ERCP: Endoscopic papillary balloon dilation; HCC: Hepatocellular carcinoma; HU: Hounsfield unit; MRCP: Magnetic resonance cholangiopancreatography; MRI: Magnetic resonance imaging; TBIL: Total bilirubin.

## Competing interests

The authors declare that they have no competing interests.
